# A glimpse of academic staff health behavior on diet type and physical activity at Austrian universities: first findings from the “Sustainably Healthy – From Science 2 Highschool & University” study

**DOI:** 10.3389/fpubh.2023.1194602

**Published:** 2023-07-06

**Authors:** Katharina C. Wirnitzer, Mohamad Motevalli, Derrick R. Tanous, Gerold Wirnitzer, Karl-Heinz Wagner, Manuel Schätzer, Clemens Drenowatz, Armando Cocca, Gerhard Ruedl, Werner Kirschner

**Affiliations:** ^1^Department of Research and Development in Teacher Education, University College of Teacher Education Tyrol, Innsbruck, Austria; ^2^Department of Sport Science, University of Innsbruck, Innsbruck, Austria; ^3^Research Center Medical Humanities, University of Innsbruck, Innsbruck, Austria; ^4^AdventureV and Change2V, Stans, Austria; ^5^Department of Nutritional Sciences and Research Platform Active Ageing, University of Vienna, Vienna, Austria; ^6^Special Institute for Preventive Cardiology and Nutrition – SIPCAN, Elsbethen, Austria; ^7^Division of Sport, Physical Activity and Health, University of Teacher Education Upper Austria, Linz, Austria

**Keywords:** lifestyle, sport, nutrition, vegan, vegetarian, plant-based, education, public health

## Abstract

**Background:**

The association between lifestyle and health status highlights the importance of assessing health-related behavior in different populations. This multidisciplinary study aimed to examine the health behavior of academic staff of Austrian colleges and universities, with a specific focus on diet types (vegan, vegetarian, omnivorous) and physical activity (PA) reports.

**Methods:**

Following a cross-sectional study design incorporating an online survey, a sample of 1,041 academics from 52 institutes (mean age: 46.4 years) provided data on sociodemographic characteristics, dietary patterns, PA behavior, and other lifestyle behaviors (smoking, alcohol intake, etc.).

**Results:**

The prevalence of vegetarian and vegan diets was 13.2 and 2.0%, respectively, and 33.2% of participants had excess body weight (BMI ≥ 25). The majority of participants (88.5%) reported regularly engaging in leisure-time PA, but 18.6% were active members of sports clubs. No difference between females and males was observed in diet type and the type of sport participation (*p* > 0.05). Participants with a mixed diet had a higher BMI than vegetarians and vegans (*p* < 0.05). Leisure-time PA participation was associated with more frequent fruit and vegetable intake (*p* < 0.05). The prevalence of smoking and alcohol intake was 13.1 and 73.5%, respectively, without any difference between dietary or sports participation subgroups (*p* > 0.05).

**Conclusion:**

The present study provides an overview of the social trends in vegan and vegetarian diets linked to health behaviors in tertiary educational settings. Findings can be used by health scientists, decision-makers, and multipliers in health and education to improve public health.

## 1. Introduction

Health is a significant aspect of human development and satisfaction. Optimal health has a pivotal role in facilitating individual and societal progress as well as enabling effective responsiveness to exogenous factors (1, 2). Despite the advances in health knowledge, there has been an increase in the prevalence of chronic health conditions worldwide, particularly non-communicable diseases (NCDs) (3, 4), which account for a staggering 74% of deaths worldwide (3). The importance of health in life highlights the responsibility of people at all levels of society to prioritize health and take action toward its sustainable improvement, requiring knowledge, competencies, and willingness to act on health-oriented goals (5, 6). Evidence indicates that related factors other than healthcare, including genetics, environment, behavior, and social circumstances (known as determinants of health), have a substantial impact (approximately 89%) on our overall state of health (7). This fact underscores the importance of adopting an integrated and holistic approach to promote health and well-being. Understanding and addressing these determinants in individual health behavior is critical in the prevention and management of most chronic diseases, including obesity, cardiometabolic disorders (such as dyslipidemia, hyperglycemia, hypertension), and psychosocial problems (such as discrimination, social isolation, and low self-esteem) (8–10). Experts suggest that physical activity (PA), nutrition, and harmful habits such as tobacco or alcohol abuse are behavioral variables that could offer valuable insight into one’s health status (11).

Regarding PA, it has been recommended by the WHO that adults should engage in a minimum of 150 min of PA per week, spread over at least three days, which is necessary to elicit beneficial health outcomes (12, 13). In fact, while adhering to or exceeding the recommended amount of PA has been associated with numerous positive health effects (13, 14), not meeting the minimum PA recommendation may negatively impact several health determinants (15). The way to carry out PA is classified mostly as leisure recreational exercise (independent of commercial providers, sports units, clubs, and federations) or participation in sports activities at a professional or amateur level (usually through membership in sports clubs). Leisure recreational exercise has been associated with better self-rated health, reduced occupational sick days, lower cardiovascular mortality and incidence of metabolic syndrome, and improved sleep quality (16–18). Participation in sports activities at a professional or amateur level has been linked to improved health and well-being, promotion of physical fitness, and increased aerobic capacity and muscular strength resulting in favorable cardiovascular health outcomes (19–21).

To optimize health outcomes, it is recommended that PA should be complemented with a healthy diet (22–24). Adopting a healthy plant-based diet is considered one of the most suitable dietary strategies for achieving sustainable lifelong health (25–28). Studies have shown that vegan and vegetarian diets may result in a healthier body mass index (BMI) and a significant reduction in all-cause mortality compared to a mixed diet (29–32). Irrespective of diet type, ensuring adequate fluid intake and the dominant consumption of nutritious food options, such as fruits, vegetables, and whole grains, is considered fundamental to a healthy diet (33–35). According to the available data, the prevalence of vegan and vegetarian diets is growing worldwide at a faster rate than expected (36, 37); however, little is known about the prevalence of vegetarian/vegan diets in different populations of academic staff/employees and their differentiated associations with other health behaviors (38–40).

Understanding the health status of any population requires analyzing the continuous interaction between PA and diet type and considering sociodemographic and individual factors that may affect health-related interactions. University professors and lecturers are prestigious role models and multipliers with great responsibility and power who are especially impactful in developing society’s sociocultural structure and fostering healthier behaviors within academic and professional environments (41, 42). University academics are at risk and face challenges in maintaining optimal health behavior, mostly due to time constraints and demanding schedules (43). In particular, tertiary faculty members have fundamentally stressful and demanding professions as they experience various stressors, including limited leisure time, inadequate rest, insufficient sleep (43, 44), which may weaken other health behaviors such as PA and dietary patterns (43, 45). This situation can put them at risk of developing a range of physical, physiological, and psychological health conditions, thus weakening their health-related quality of life (46–48).

Given their influential role in shaping the attitudes and actions of their students, colleagues, and wider social networks (42, 49), investigating health behavior of tertiary college/university (tertiary level) lecturers, researchers, and professors (from here they will be referred to with the umbrella term “academic staff”) within the context of long-term sustainable health can help to identify potential areas for establishing policies and guidelines aimed at promoting healthy behavior in educational settings. Promoting healthy behavior among academic staff seems to be not only beneficial for educational individuals but also for the overall success of public health strategies and efficient long-term outcomes. Existing studies indicate that the prevalence of excess body weight among academic staff is high (47, 50), nearly two-thirds do not meet the recommended PA level (51, 52), and their health-related quality of life is poor (53, 54). In this regard, however, interventions targeting academic staff health behavior were proven effective (45).

There is a knowledge gap in the literature on the association between physical exercise behavior and diet types (particularly discriminating omnivorous vs. vegetarian and vegan diets) among academic staff, except for one recent report on secondary school teachers from our laboratory (55). Thus, exploring lifestyle health behavior focusing on dietary subgroups and analyzing the possible associations with PA engagement among academic staff seems of utmost importance. Therefore, the present study aimed to examine the current health behaviors of academic staff in Austria based on a large sample, with a particular emphasis on the prevalence of vegetarian and vegan diets and the linkage with PA engagement and sociodemographic determinants, including age, sex, BMI, and living environment.

## 2. Materials and methods

### 2.1. Study design and sample

The “*Sustainably Healthy – From Science 2 Highschool & University*” study is a comprehensive research project that uses a multidisciplinary approach and a multilevel cluster sampling method to investigate the health and well-being of academic staff in Austria. It is a in direct continuation of a previous study conducted on Austrian secondary school populations (including pupils and their teachers and headmasters) (56). The Federal Ministry of Education, Science, and Research of Austria (BMBWF; Department 1/7 – School and University Sports) has supported this study. A total of 52 out of 102 Austrian colleges/universities participated in the study, and approval from their ethics board was obtained before data collection. Further methodological information is available in the study protocol (57).

The study intended to attract as many individuals as possible to participate from the potential sample of 69,310 eligible academic staff. The dean boards at all Austrian colleges/universities were contacted in order to provide relevant preliminary information about the study’s purpose, procedures, and instructions and to invite their academic staff to contribute by filling out an online survey.

### 2.2. Study procedure

An online survey (LimeSurvey version 3.25.15) was available in German and data collection was based on self-reported information. Participants were able to provide data between the 5th of April and the 31st of July 2021, and they provided written informed consent after getting familiar with the study’s goals and procedures. Taking part was voluntary and anonymous, and they could withdraw without giving a reason at any time. Participants could complete the standardized survey in approximately 20 min using their smartphone, tablet, or PC/laptop and following a web link with an encrypted interface.

**Figure 1:**
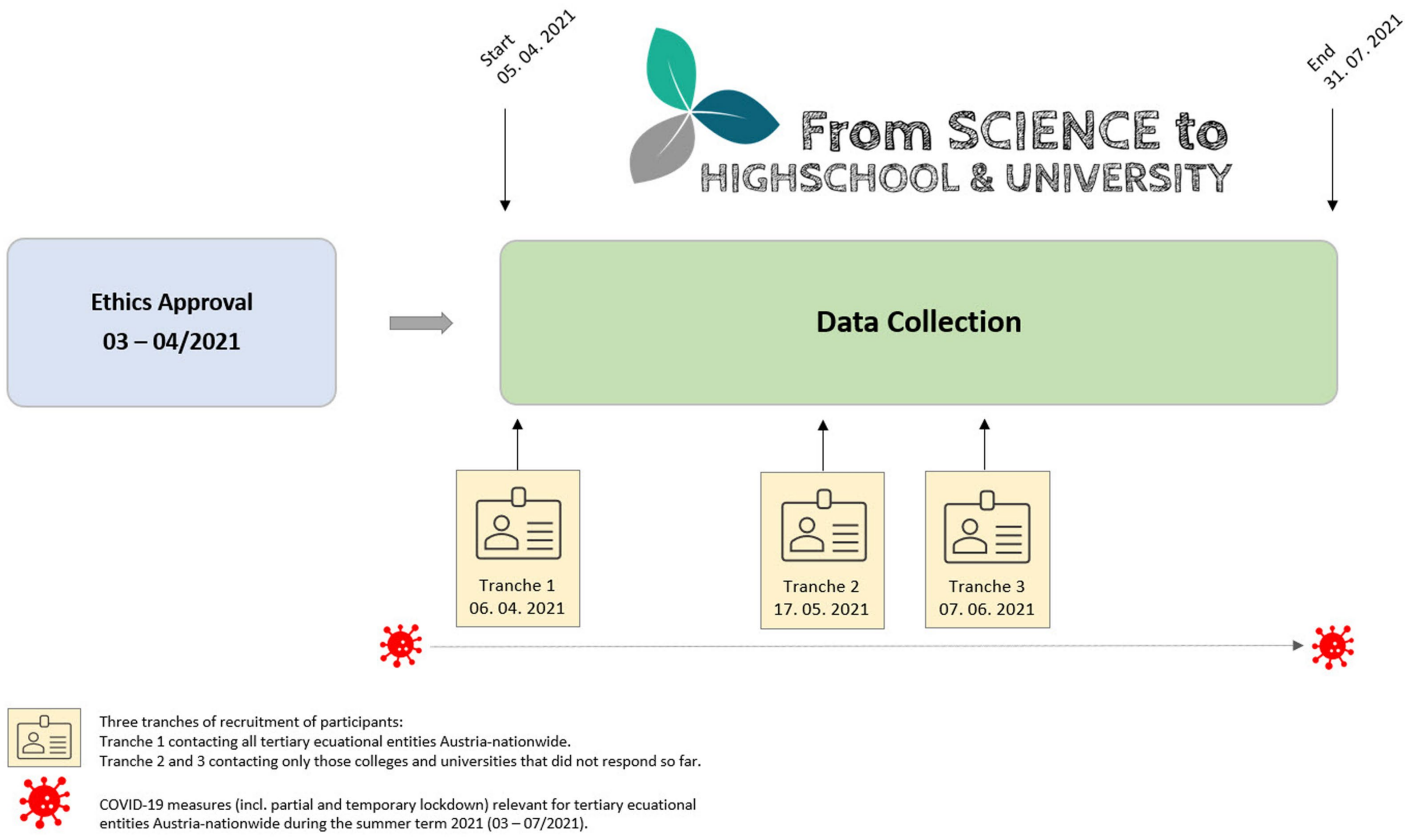
The study procedure and timeline from ethics approval issuance to data collection completion.

The survey had six sections, consisting of personal information (Part A), sports and exercise (Part B), nutrition (Part C), health and well-being (Part D), COVID-19 pandemic (Part E), and miscellaneous (Part F) (57). The survey included control questions to ensure data reliability by identifying inconsistent responses. The study procedure and timeline are presented in Figure 1.

### 2.3. Measures, variables, and classifications

The online survey gathered information on a variety of factors, including socio-demography (age, sex, nationality, federal state, living environment, marital status, and highest academic level), anthropometry (body weight and height), professional status (subject area, employment status: full-time/part-time, and type of tertiary educational entity), dietary information (current adherence to a specific kind of diet, type and frequency of fluid intake, and frequency of fruit and vegetable intake), PA (sports type, duration, frequency, participation in competitions, leisure-time PA, sports club membership, etc.), and other lifestyle factors (including alcohol consumption and smoking).

At the end of data collection, a total number of 1,043 college/university academic staff provided complete data sets, which corresponds to approximately 1.5% of the eligible sample size in Austria. Participants with missing or conflicting data were removed from the study sample, resulting in a final sample size of 1,041 adults after data clearance. This sample consisted of individuals from all nine federal states of Austria.

**Figure 2:**
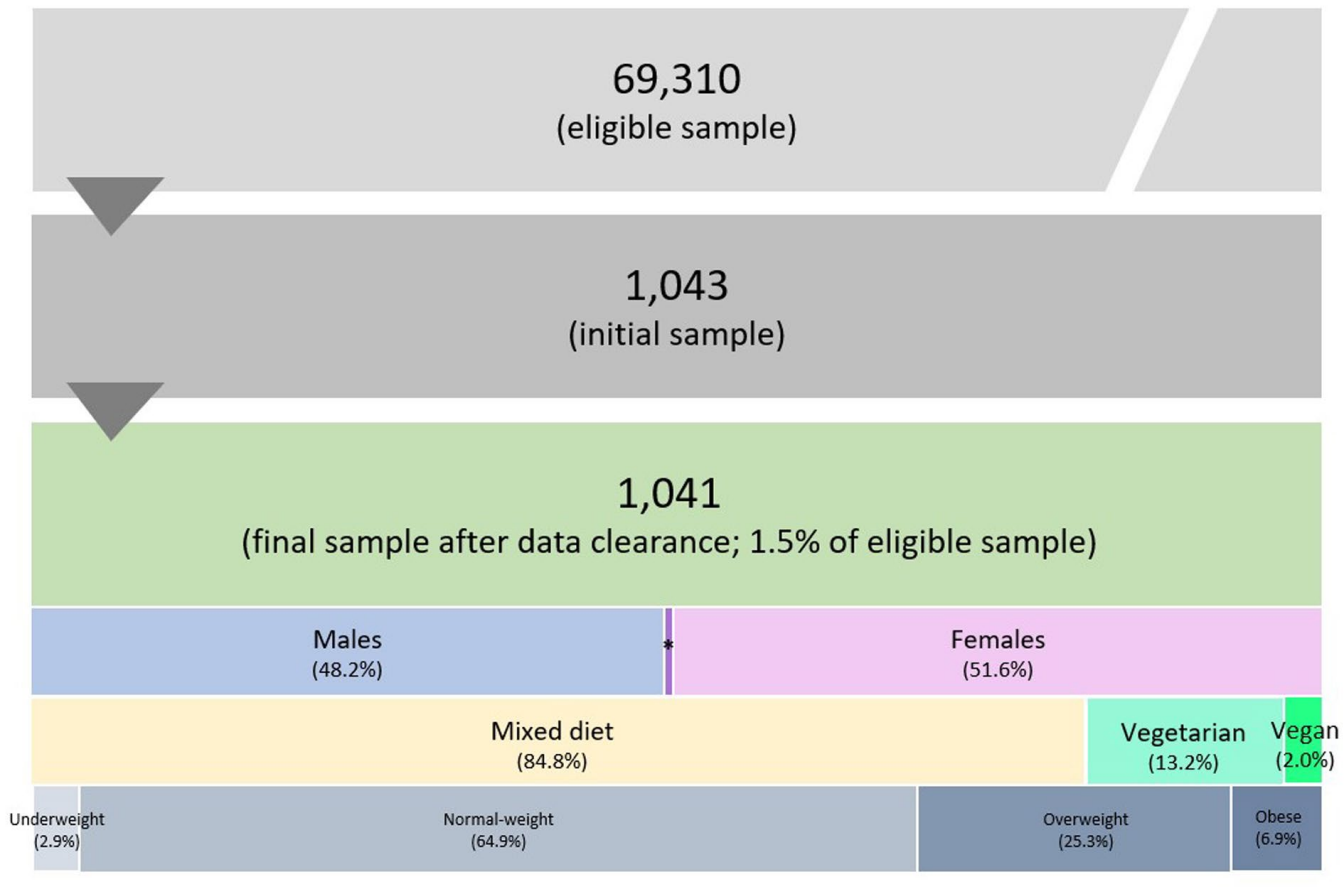
Sample size and classification of participants based on sex, diet type, and BMI. *Diverse population, representing 0.2% of the final sample size.

BMI was calculated using height and body weight values, and participants were assigned to one of four BMI subgroups (underweight, normal weight, overweight, and obese) based on the WHO’s cut-points (58, 59). Participants were also classified into one of three dietary subgroups: vegetarian (no meat and processed meat, meat products, fish, or shellfish, but consumption of dairy, eggs, and honey), vegan (no intake of any animal-sourced foods or ingredients), or mixed diet (no dietary restrictions on food sources) (27). Figure 2 displays the sample size and classification of participants.

### 2.4. Statistical analysis

Statistical analyses were performed using SPSS version 26.0 (SPSS Inc., IBM Corp., Armonk, NY, USA). Descriptive statistics were used for exploratory analysis, and continuous data are presented as the mean ± standard deviation (SD), while nominal data are reported as prevalence/percentage. Multivariate analysis of variance (MANOVA) was utilized to examine the association of sex, employment status (full-time/part-time), diet type, and sports participation with anthropometric characteristics and age. Differences in sex, living environment, nationality, and health behaviors by diet and sports participation were assessed using chi-square tests. The statistical significance level was set at *p* ≤ 0.05.

## 3. Results

From a total number of 1,041 participants, 71.1% lived in urban areas, 81.9% were Austrian, and 65.9% were fully employed at a college and/or university. The majority of non-Austrian participants came from countries within the European Union (91.0%) with Germany (66.0%) and Italy (11.2%) representing the most common nationalities. Table 1 shows the distribution of participants by living situation, nationality, and employment status.

**Table 1:** Sample distribution by sex and employment status.

	Total (*n*)	Male *n* (%)	Female *n* (%)	Diverse *n* (%)	Full time *n* (%)	Part time *n* (%)
	1,041	502 (48.2)	537 (51.6)	2 (0.2)	686 (65.9)	355 (34.1)
Living Environment
Urban	740	363 (49.1)	375 (50.7)	2 (0.3)	499 (67.4)	241 (32.6)
Rural	301	139 (46.2)	162 (53.8)	0 (0.0)	187 (62.1)	114 (37.9)
Nationality
Austrian	853	414 (48.5)	437 (51.2)	2 (0.2)	549 (64.4)	304 (35.6)
Other nationalities	188	88 (46.8)	100 (53.2)	0 (0.0)	137 (72.9)	51 (27.1)
Federal State of Institution
Burgenland	40	19 (47.5)	21 (52.5)	0 (0.0)	18 (45.0)	22 (55.0)
Carinthia	12	2 (16.7)	10 (83.3)	0 (0.0)	8 (66.7)	4 (33.3)
Lower Austria	38	18 (47.4)	20 (52.6)	0 (0.0)	30 (78.9)	8 (21.1)
Salzburg	67	30 (44.8)	37 (55.2)	0 (0.0)	36 (53.7)	31 (46.3)
Styria	61	18 (29.5)	43 (70.5)	0 (0.0)	35 (57.4)	26 (42.6)
Tyrol	281	123 (43.8)	158 (56.2)	0 (0.0)	189 (67.3)	92 (32.7)
Upper Austria	120	45 (37.5)	75 (62.5)	0 (0.0)	75 (62.5)	45 (37.5)
Vienna	402	238 (59.2)	162 (40.3)	2 (0.5)	279 (69.4)	123 (30.6)
Vorarlberg	20	9 (45.0)	11 (55.0)	0 (0.0)	16 (80.0)	4 (20.0)

### 3.1. Anthropometric characteristics

No age difference was observed between full-time and part-time employment status. Participants with full-time employment, however, had a significantly higher BMI, which resulted in a higher prevalence of overweight compared to those with a part-time employment at a tertiary institution (*p* < 0.01). The prevalence of obesity and underweight did not differ by employment status. While male participants had a higher BMI than females, the prevalence of underweight and normal weight in females and overweight and obesity in males were higher than the opposite sex. Table 2 shows the anthropometric characteristics across the total sample and by sex and employment status (full time vs. part time). Additional anthropometric data by federal state and study level (separately for urban and rural areas) are available in the Appendix as Tables A1, A2.

**Table 2:** Anthropometric characteristics by sex and employment status.

	Total	Male	Female	Diverse	Full time	Part time
Age (years)	46.4 ± 11.5	47.9 ± 12.3	45.0 ± 10.5	47.5 ± 16.3	46.5 ± 10.6	46.3 ± 13.1
Urban	46.0 ± 11.9	47.5 ± 12.7	44.6 ± 10.8	47.5 ± 16.3	46.0 ± 10.8	46.1 ± 13.9
Rural	47.4 ± 10.5	49.1 ± 11.1	46.0 ± 9.7	**–**	47.9 ± 9.9	46.7 ± 11.3
Body weight (kg)	72.9 ± 14.2	81.0 ± 12.2	65.3 ± 11.5	65.4 ± 8.8	74.1 ± 14.1	70.6 ± 14.1
Urban	72.6 ± 14.3	80.9 ± 12.5	64.6 ± 11.0	65.4 ± 8.8	73.8 ± 14.1	70.1 ± 14.2
Rural	73.6 ± 14.0	81.5 ± 11.2	66.8 ± 12.4	**–**	74.7 ± 14.0	71.7 ± 13.7
Height (cm)	173.9 ± 9.0	180.3 ± 7.0	167.9 ± 6.0	169.5 ± 2.1	174.5 ± 9.1	172.7 ± 8.6
Urban	173.8 ± 8.9	180.0 ± 7.1	167.8 ± 5.9	169.5 ± 2.1	174.4 ± 9.1	172.6 ± 8.4
Rural	174.1 ± 9.1	181.1 ± 6.7	168.0 ± 6.0	**–**	174.9 ± 9.1	172.9 ± 9.1
BMI (kg/m^2^)	24.0 ± 3.8	24.9 ± 3.5	23.2 ± 3.9	22.8 ± 3.6	24.2 ± 3.7	23.6 ± 3.9
Urban	23.9 ± 3.7	25.0 ± 3.5	22.9 ± 3.6	22.8 ± 3.6	24.2 ± 3.6	23.4 ± 3.8
Rural	24.2 ± 3.9	24.8 ± 3.3	23.7 ± 4.4	**–**	24.4 ± 3.9	23.9 ± 3.9
Underweight (%)	2.9	1.2	4.5	–	2.8	3.1
Urban	3.1	1.4	4.8	–	2.4	4.6
Rural	2.3	0.7	3.7	–	3.7	0.0
Normal weight (%)	64.9	56.8	72.6	50.0	63.3	68.2
Urban	65.8	56.5	74.9	50.0	64.5	68.5
Rural	62.8	57.6	67.3	–	59.9	67.5
Overweight (%)	25.3	34.3	16.8	50.0	27.0	22.0
Urban	24.3	34.2	14.7	50.0	26.1	20.7
Rural	27.6	34.5	21.6	–	29.4	24.6
Obese (%)	6.9	7.8	6.1	–	7.0	6.8
Urban	6.8	8.0	5.6	–	7.0	6.2
Rural	7.3	7.2	7.4	–	7.0	7.9

### 3.2. Participation in PA/sports

From the total sample, 88.4% of participants reported regular PA participation during their leisure time, and 18.6% of them were active members of sports clubs. Average sports participation was 3.3 ± 1.6 days per week across participants who reported regular engagement with sports. Sports participation did not differ by sex, employment status, living environment, or nationality. There were also no differences in anthropometric characteristics between active club sports members and non-members. Regular engagement in leisure-time PA, on the other hand, was associated with a lower BMI (*p* < 0.01). In particular, the average BMI of those not reporting any leisure-time PA was in the overweight category while the average BMI of participants reporting regular leisure-time PA was in the normal range. Accordingly, the prevalence of overweight and obesity was higher in participants who did not report any sports participation compared to those with regular engagement in leisure-time PA (*p* < 0.01). The distribution of participants by PA/sports participation is displayed in Table 3, while Table 4 displays anthropometric characteristics by PA/sports participation.

**Table 3:** Differences in sex, employment status, living environment, and nationality by type of PA/sport participation.

	Leisure-time PA *N* (%)	Club Sports *N* (%)	PA/Sport days per week* Mean ± SD
Total Sample	921 (88.5)	194 (18.6)	3.3 ± 1.6
Male	435 (86.7)	112 (22.3)	3.2 ± 1.6
Female	484 (90.1)	81 (15.1)	3.4 ± 1.6
Diverse	2 (100.0)	1 (50.0)	4.0 ± 1.4
Employment Status
Full time	611 (89.1)	135 (19.7)	3.3 ± 1.6
Part time	310 (87.3)	59 (16.6)	3.3 ± 1.5
Living Environment
Urban	651 (88.0)	128 (17.3)	3.3 ± 1.6
Rural	270 (89.7)	66 (21.9)	3.3 ± 1.6
Nationality
Austria	759 (89.0)	162 (19.0)	3.3 ± 1.6
Other	162 (86.2)	32 (17.0)	3.2 ± 1.6

* Only participants who reported regular PA/sports participation are included.

**Table 4:** Differences in anthropometric characteristics by type of PA/sport participation.

	Leisure-time PA		Club sports	
	Yes	No	Yes	No
Age (years)	46.4 ± 11.6	46.6 ± 11.0	46.5 ± 11.2	46.4 ± 11.6
Height (cm)	173.7 ± 8.9	174.9 ± 9.6	174.7 ± 8.9	173.7 ± 9.0
Body Weight (kg)^1^	72.0 ± 13.5	80.0 ± 16.9	73.0 ± 13.4	72.9 ± 14.4
BMI (kg/m^2^)^1^	23.7 ± 3.5	26.1 ± 5.0	23.8 ± 3.2	24.1 ± 3.9
Underweight (%)	3.0	1.7	2.6	3.0
Normalweight (%)^1^	67.2	47.5	68.6	64.1
Overweight (%)^1^	24.3	32.5	24.2	25.5
Obese (%)^1^	5.4	18.3	4.6	7.4

1 Significant difference between those with and without leisure-time PA (*p* ≤ 0.01).

### 3.3. Diet type

Based on the reported dietary intake, 84.8% of participants consumed a mixed diet, while 13.2 and 2.0% were vegetarian and vegan, respectively. The prevalence of vegan and vegetarian diets was higher in women compared to men. There were no significant differences between diet types by employment status, living environment, or nationality. Participants consuming a mixed diet were significantly older than those reporting a vegetarian or vegan diet (*p* < 0.01). A vegetarian and vegan diet was also associated with a significantly lower BMI along with a lower prevalence of overweight and obesity compared to those reporting a mixed diet (*p* < 0.01). Even though the average BMI of participants reporting a vegan diet was similar to those reporting a vegetarian diet, there were no significant differences in anthropometric characteristics between participants with mixed diet and vegan diet, which may be attributed to the small number of vegans in the study population. The distribution by diet type prevalences along with sociodemographic and anthropometric characteristics is displayed in Tables 5, 6.

**Table 5:** Differences in sex, study level, living environment, and nationality by diet type.

	Mixed diet *N* (%)	Vegetarian *N* (%)	Vegan *N* (%)
Total sample	883 (84.8)	137 (13.2)	21 (2.0)
Male	446 (88.8)	49 (9.8)	7 (1.4)
Female	435 (81.0)	88 (16.4)	14 (2.6)
Diverse	2 (100)	0 (0.0)	0 (0.0)
Employment status
Full time	588 (85.7)	85 (12.4)	13 (1.9)
Part time	295 (83.1)	52 (14.6)	8 (2.3)
Living environment
Urban	620 (83.8)	106 (14.3)	14 (1.9)
Rural	263 (87.4)	31 (10.3)	7 (2.3)
Nationality
Austria	736 (86.3)	103 (12.1)	14 (1.6)
Other	147 (78.2)	34 (18.1)	7 (3.7)

**Table 6:** Differences in anthropometric characteristics by diet type.

	Mixed diet	Vegetarian	Vegan
Age (years)^1,2^	47.4 ± 11.3	41.7 ± 11.2	36.7 ± 10.5
Height (cm)^1^	174.3 ± 8.9	172.0 ± 8.7	169.9 ± 10.8
Body Weight (kg)^1^	74.0 ± 14.3	66.8 ± 11.6	65.4 ± 12.2
BMI (kg/m^2^)^1^	24.3 ± 3.9	22.5 ± 2.8	22.6 ± 3.1
Underweight (%)	20 (2.3)	9 (6.6)	1 (4.8)
Normal Weight (%)^1^	555 (62.9)	105 (76.6)	16 (76.2)
Overweight (%)^1^	239 (27.1)	20 (14.6)	4 (19.0)
Obese (%)^1^	69 (7.8)	3 (2.2)	0 (0.0)

^1^Significant difference between mixed diet and vegetarian diet (*p* ≤ 0.01). ^2^Significant difference between mixed diet and vegan diet (*p* ≤ 0.01).

### 3.4. Health behavior by PA/sport participation and diet type

Leisure-time PA participation was associated with a higher prevalence of daily fruit and vegetable consumption, while there was no significant difference in fruit and vegetable consumption between active members in club sports and those not participating in club sports. The prevalence of daily fruit and vegetable consumption also increased with the number of days participants engaged in PA/sports (*p* for trend <0.01). Additionally, the prevalence of participants with a fluid intake above 2 L/day increased as the number of days with PA/sports engagement increased (*p* < 0.001). 73.3% of the participants reported water as their most commonly consumed fluid and there was no difference by type of PA/sports participation. Similarly, the prevalence of those drinking alcohol or smoking did not differ by type of PA/sports participation. Table 7 displays the association between type and frequency of PA/sports participation and diet-related health behavior.

**Table 7:** Dietary habits by PA/sports participation presented as prevalence (%).

	Leisure-time PA		Club Sports		PA/Sport days per week*		
	Yes	No	Yes	No	0–1 days	2–4 days	5–7 days
Daily Fruit^1,2^	64.8	43.3	62.9	62.2	47.9	66.0	66.5
Daily vegetable^1,2^	81.4	67.5	76.3	80.2	67.4	82.2	83.8
Fluid Intake (> 2 L/day)^2^	39.1	32.5	45.4	36.7	33.0	36.5	50.3
Water as most common drink	74.6	66.7	75.3	73.3	68.8	73.9	78.5
Alcohol	73.7	71.7	77.8	72.5	73.5	75.6	66.5
Smoking	12.2	20.0	10.3	13.7	17.7	12.1	11.0

*Only participants who reported regular PA/sports participation are included. ^1^Significant difference between those with and without leisure-time PA (*p* < 0.01). ^2^Significant difference between frequencies of PA/sport participation (*p* < 0.01).

The prevalence of participants reporting regular engagement in PA/sports did not differ significantly across dietary patterns (Table 8). Participants reporting a vegetarian diet, however, reported a more frequent sports participation compared to those with a mixed diet (3.8 ± 1.6 vs. 3.2 ± 1.5 days/week; *p* < 0.01). There was no difference by diet type in fluid intake or the prevalence of participants reporting water as most commonly consumed fluid. The prevalence of participants drinking alcohol, however, declined from those with a mixed diet to those reporting a vegetarian and vegan diet (*p* for trend = 0.01). Despite the fact that none of the participants reporting a vegan diet smoked, no significant differences were observed for smoking across dietary patterns. Additional information on PA/sports participation, eating behaviors, alcohol consumption, and smoking by federal states and living environment is provided in the Appendix as Tables A3, A4.

**Table 8:** Health behaviors by dietary pattern presented as prevalence (%).

	Mixed diet	Vegetarian	Vegan
Leisure-time PA participation	87.7	94.2	85.7
Club sports participation	19.5	14.6	9.5
Fluid Intake (> 2 L/day)	38.2	38.7	42.9
Water as most common drink	73.6	74.5	71.4
Alcohol	75.1	65.7	57.1
Smoking	13.3	13.9	0.0

## 4. Discussion

This study aimed to examine for the first time the health-related behavior of academic staff focusing on the prevalence of vegan, vegetarian, and mixed diets linked to PA patterns nationwide across Austria. In addition, it is the first attempt to investigate health behavior emphasizing the dual approach of sustainable health by a large and representative sample of academic staff in Austria (*n* = 1,041, from 52 tertiary entities) analyzing the potential associations with sociodemographic factors (including sex, age, BMI, nationality, living environment, and employment status). The most considerable findings were: (i) over one-third of the participants (33.2%) were overweight or obese; (ii) 9 of 10 participants reported to have regular leisure-time PA, but less than 20% were active members in sports clubs; (iii) the majority of the participants (84.8%) reported to follow a mixed diet, while the prevalence of vegetarian and vegan diets were 13.2 and 2.2%, respectively; (iv) leisure-time PA participation was associated with a lower BMI and lower prevalence of overweight/obesity, while there were no differences in anthropometric characteristics by club sports participation; (v) a mixed diet was associated with a higher BMI and higher prevalence of overweight and obesity compared to vegetarian and vegan diets; and (vi) leisure-time PA participation (but not club sports participation) was associated with a more frequent fruit and vegetable intake.

An overview of the findings in this study suggests that academic staff of Austrian colleges and universities have a general tendency toward a healthier lifestyle (considering lower rates of smoking, alcohol consumption, physical inactivity, and obesity) compared to the general populations in Austria (60, 61). This observation aligns with the conclusion of a previous health study in Austria, which reported that teachers generally have good-to-excellent health statuses (62). Overall, good health is closely linked to action competence and personality development with positive attitudes toward healthy lifestyles and behaviors (63). Research has shown that poor behavioral patterns, especially unhealthy eating habits and insufficient PA, are the primary risk factors contributing to poor health and well-being (64). The Organization for Economic Cooperation and Development (OECD) has reported that Austrians have a life expectancy similar to that of other European Union countries (81.3 vs. 81.0 years, respectively); however, compared to their European counterparts, people living in Austria have fewer years of disability-free living (57 years vs. the EU average of 64 years) and experience more years with chronic illnesses or disabilities (64).

The present study showed that overweight/obesity was considerably more prevalent in male participants (42.1%) compared to females (22.9%). Consistent with the present findings, results from two comparable Austrian surveys on schoolteachers showed that the prevalence of overweight/obesity was remarkably higher in males (40.6 and 46.5%) than in females (14.7 and 29.2%) (55, 65). According to a Spanish study, a greater proportion of male and female university professors were found to be overweight or obese (64.5% of males and 36.9% of females) (43). While the prevalence of overweight/obesity is higher among the general population of Austrian adults compared to academics, the sex difference between males and females has been reported to be much smaller (66). A review study indicates that there are either no or minimal sex differences in the overall prevalence of excess body weight in high-income countries (67). Although this social aspect may partly explain the present study’s findings, it is important to acknowledge that females are typically more health-conscious than males (68–70), and this tendency is likely more pronounced among academic populations (71). Our findings indicate that females (4.5%) have a higher prevalence of being underweight compared to males (1.2%), which is consistent with previous studies (55, 65). Although the prevalence of underweight has decreased in the general population over the past two decades, the percentage of underweight individuals is marginally higher in females (9.7%) than in males (8.8%) globally (72), and various factors, including dietary-related causes (73), may contribute to the occurrence of underweight. Colleges and universities can introduce initiatives such as providing healthy food options on campus (e.g., public catering, vending machines) and encouragements for active mobility and commuting. Additionally, offering health-related programs that involve BMI assessments, personalized counseling, and group support could be effective in assisting faculty members in attaining and maintaining a healthy weight status.

Evidence indicates that Austrian adults typically engage in PA at a level that exceeds the recommended amount (64, 74, 75). To some extent, this finding is in line with the present study’s results regarding consistent participation in leisure-time PA where 90.1% of females and 86.7% of males had an active lifestyle, with PA/sport frequency of 3.4 days per week for females and 3.2 days per week for males. However, the rate of club sports participation was only 15.1% in females and 22.3% in males. While the present study did not gather data on PA duration, a minimum of 150 min moderate-to-vigorous PA, or 75 min vigorous PA, over 4–5 days per week has been recommended as the optimal duration and frequency of PA (76, 77). The present findings suggest that the participation rate in leisure-time PA is similar between academic staff and secondary schoolteachers in Austria (55), with rates of 88.5 and 88.7%, respectively. In general, it is notable that PA habits can be influenced by environmental factors, such as the presence of recreational sport facilities (78) and/or natural landscapes (79). According to three similar studies on university academics, the prevalence of physical inactivity ranged between 42 and 69% (51, 52, 80), which is higher than in the present study. These variations may be attributed to several factors, including variations in the methodological strategies conducted to measure physical inactivity, the absence of a theoretical framework to explain sustained behavior change, and the focus on distinct populations in diverse settings (81). As a result, when utilizing these data to make conclusions about the extent of physical inactivity among academic staff, it is crucial to consider the differences in populations and assessment techniques. In general, academic staff are vulnerable to inadequate PA levels, as they typically engage in prolonged periods of sedentary behavior even during their leisure time (82). Therefore, it seems essential for educational settings to offer interventions, such as on-campus fitness centers, enlarge in-campus sports offers for affiliated tertiary employees, or incentives to increase PA levels.

It was discovered that there is a notable association between differences in BMI and engagement in leisure-time PA (but not club sports). The results indicated that individuals who are regularly engaged in PA have an average BMI of 23.7 kg/m^2^, which is within the normal range classified as healthy, while those who do not participate in PA have an average BMI of 26.1 kg/m^2^. Accordingly, the prevalence of overweight/obesity was significantly different among those with (29.7%) and without (50.8%) leisure-time PA. Consistent with the present findings, previous reports indicate that engaging in leisure-time PA contributes to weight management, leading to lower rates of obesity and overweight (83). Therefore, it can be recommended that participating in leisure-time PA on a regular basis, even if it falls below the internationally recommended level, can be a useful approach for weight management. In this regard, active mobility and commuting to different places (workplace, home, shopping, etc.) is known to be an essential component of daily PA levels that may gradually lead to a better body weight profile (84). The findings of a systematic review indicate that active commuting can provide health benefits similar to those of moderate exercise training (85). Dietary habits are also known to play a crucial role in body weight and health status. Participants who were engaged in regular leisure-time PA consumed significantly more fruits (64.8% vs. 43.3%) and vegetables (81.4% vs. 67.5%) on a daily basis compared to non-active academic staff; however, there was no significant difference in dietary habits between those who participated in sports clubs and those who did not. Consistently, available evidence shows a close connection between PA/sport engagement and the consumption of healthy food choices (22, 86). However, the findings on dietary intake in this study may not be linked to general diet types, where omnivores, vegetarians, and vegans had similar rates of PA engagement both during leisure time and through participation in club sports. This finding is partially inconsistent with the data on younger populations (87, 88), where a significant association between diet type and rates of PA engagement has been reported.

The dietary profile of the participants in the present study showed a prevalence of 15.4% of vegetarian and vegan diets (13.2 and 2.0%, respectively) with higher fractions of females than males following a vegetarian (16.4% vs. 9.8%) or vegan (2.6% vs. 1.4%) diets. Global data show that the prevalence of both vegan and vegetarian diets is increasing (89, 90), and currently, about 10% of European adults follow a vegan or vegetarian diet (91). The higher prevalence of plant-based diets in the present study may be partially justified by the generally higher health consciousness of academic populations (70) or the greater prevalence of vegetarian kinds of diet in German-speaking countries compared to the European average (92, 93). However, data from the same laboratory (55) show that the prevalence of vegan/vegetarian diet in Austrian school teachers is lower than their colleagues at the tertiary education level in the present study (10.9% vs. 15.2%, respectively). However, Austrian college/university students are even more likely to follow vegetarian diet types than their lecturers and professors (28.8% vs. 15.2%, respectively) (94), which is in line with the high prevalence of vegetarian and vegan diets recently observed among secondary school pupils in Austria (87). Taken together, these findings can be partly explained by reports suggesting that younger individuals are more inclined toward adopting more sustainable vegetarian diet types in comparison to older adults (95, 96). While further research is needed to elucidate the variations in behavioral patterns associated with different kinds of diet, it is well established that vegetarian and vegan dietary patterns are both adequate for providing individuals with all the essential nutrients necessary for healthy growth and development (26), and plays a protective role against several diseases, including cancer and diabetes (27, 28).

The present study shows that omnivorous participants have a higher BMI than those who follow vegetarian or vegan diets. Consequently, the prevalence of overweight and obesity is about two times higher in omnivores than in vegetarian and vegan participants. Previous research has also consistently reported that vegans have a lower body weight and BMI than omnivores, resulting in a lower prevalence of overweight and obesity among individuals following a vegetarian diet type (97, 98). A randomized controlled trial examining the impact of different diet types on body weight changes in adults found that vegetarian/vegan diets were more effective than mixed diets for weight loss purposes (99). A longitudinal study of a large adult cohort where dietary habits and anthropometric data were surveilled over 6.5 years consistently showed a significant correlation between the consumption of animal proteins and long-term weight gain (100). The study on secondary school teachers in Austria reported similar findings regarding BMI across different diet types, with vegetarians and vegans having a lower BMI compared to omnivores (55). One possible explanation for these findings is that individuals who adhere to vegetarian or vegan diets tend to be more health-conscious in general (101). It is important to note that regardless of the type of diet, the quality of the diet and its components (which were not examined in the present study) have been shown to be strong predictors of both health and body weight management (33, 102); thus, it is important to cautiously interpreting study findings.

Results from the present study showed that the prevalence of smoking and alcohol intake was 13.1 and 73.5%, respectively, without any difference between diet types and kind of PA engagement (leisure time and club sports). Compared to findings of our previous investigation on school teachers in Austria (55), it seems that academic staff drink less alcohol (73.5% vs. 81.5%) but smoke slightly more (13.1% vs. 11.5%). Another study demonstrated that educators had a reduced occurrence of smoking on a daily basis compared to the general public (12.6% vs. 23.3%), with the majority of teachers quitting as they age (65, 71). In this regard, evidence indicates that there is significant association between psychoactive substance intake (in terms of consuming alcohol, tobacco, and cannabis) and occupational stress among academic staff (103), which may potentially cause several health conditions. Therefore, tertiary educational entities are encouraged to invest in health behavior concerning their human capital by implementing health-promoting interventions and offers, especially in academic staff with extensive working hours.

The present study has limitations necessary to be mentioned: (i) the cross-sectional design that restricts the ability to establish cause-and-effect relationships; (ii) the possibility of socially desired misreporting, such as over-reporting of the consumption of healthy food items, duration of PA/sport engagement, and under-reporting of body weight, and rates of alcohol intake or smoking. However, to enhance the reliability and accuracy of the data, the survey included multiple control questions at various points and sections, which aimed at uncovering conflicting responses and minimizing the possibility of misreporting (conflicting data identified were removed or revised from the analyses accordingly); (iii) the PA assessment method used in the study did not offer comprehensive information on exercise habits, particularly the intensity and duration of PA; (iv) while the Austria-wide sample permits the generalization of the study results to Austria, as well as countries with similar culture and geography such as Germany and Switzerland, other factors such as socio-environmental characteristics, culture, and varying school systems around the world may limit the representativeness of the lifestyle-related outcomes; (v) the questionnaire was not validated due to the fact that the majority of variables in the survey were constructed using single questions, which originates from some pre-existing limitations, including a low number of discrimination points and the impossibility to assess internal consistency (104). However, it has been noted that single-item questionnaires can be considered valid and may help to overcome the limitations associated with longer surveys (105, 106); (vi) the distribution of dietary groups was unbalanced, primarily due to the lower prevalence of vegetarian and vegan populations compared to omnivores, which is a commonly observed phenomenon in similar prevalence studies. In spite of this inherent limitation (107), it is noteworthy that the sample size was statistically sufficient to generate meaningful insights regarding the relationship between the independent and dependent variables. Despite these limitations, the present multidisciplinary study can be regarded as the novel investigation of the prevalence of different kinds of diet among academic staff in Austria and the associations with other health behaviors, especially of PA levels.

## 5. Conclusion

The present nationwide study examined the health behaviors of 1,041 academic staff from 52 Austrian colleges and universities for the first time. Overall, the sample population exhibited healthier lifestyle behaviors (in terms of lower BMI, healthier dietary behaviors, increased leisure-time PA, and less smoking) than the general population, as reported by comparable investigations. Higher levels of education and academic qualification, advanced individual capabilities, and social advantages of academic staff may contribute to their healthier lifestyles. However, given the importance of academic staff as role models in educating and guiding future generations, the findings here emphasize the need for ongoing efforts to promote healthier individual lifestyle choices in colleges and universities. The present study provides valuable insights into current health and lifestyle behaviors, particularly the “healthy eating and active living” dual approach of sustainable and lifelong health, and can guide reflections as well as updates to health-related recommendations. Additionally, the study identifies social trends in health behavior in tertiary educational settings, which can help colleges and universities to offer healthy and sustainable food options as well as PA, sports & exercise through mobility opportunities. Health behavior monitoring is necessary to understand lifelong and sustainable trajectories, and improving health literacy through educational policies can enhance public health and well-being. The study’s findings can be used by health specialists, policy and decision-makers, and educators as a starting point for future measures and interventions to improve public health by targeting the individual health of academic staff, the multipliers and role models.

## Data availability statement

The datasets presented in this article are not readily available because due to Austrian data security law, and additionally to the requirements of all the tertiary educational entities considering data on students and employees, it is not applicable. Requests to access the datasets should be directed to katharina.wirnitzer@ph-tirol.ac.at.

## Ethics statement

This study was conducted in accordance with the medical professional codex, the Helsinki Declaration as of 1996, Data Security Laws and good clinical practice guidelines. The study protocol was approved by both the ethics board of the Rectorate of the University College of Teacher Education Tyrol (PHT-HSa-17-Z1.8-5n_4927; March 22, 2021) and the “Board for Ethical Questions in Science of the University of Innsbruck,” Vice-Rectorate for Research (Certificate of good standing, 22/2021; April 6, 2021). Written informed consent was obtained from all participants involved in the study. Participation in the study was voluntary and could be terminated at any time without providing reason or negative consequences.

## Author contributions

KW, GR, WK, CD, and K-HW: conceptualization and study design. KW and CD: methodology and formal analysis. KW, CD, and MM: writing original draft preparation. KW, MM, DT, MS, CD, GR, AC, and WK: critical review and editing. GW: technical support. All authors have read and agreed to the published version of the manuscript.

## Funding

This Austria nationwide study is funded by the TWF (Tiroler Wissenschaftsförderung; reference number: F.30976/6-2021). However, the TWF was and is still not involved in the study procedures, and thus, there is and will be no impact from the funding agency on the study design, conduction and data collection, data analysis, presentations, and/or publication of the results.

## Conflict of interest

The authors declare that the research was conducted in the absence of any commercial or financial relationships that could be construed as a potential conflict of interest.

## Publisher’s note

All claims expressed in this article are solely those of the authors and do not necessarily represent those of their affiliated organizations, or those of the publisher, the editors and the reviewers. Any product that may be evaluated in this article, or claim that may be made by its manufacturer, is not guaranteed or endorsed by the publisher.
