# Supplementary material for: A glimpse of academic staff health behavior on diet type and physical activity at Austrian universities: first findings from the “Sustainably Healthy – From Science 2 Highschool & University” study
Source: Front Public Health. 2023 Jul 6;11:1194602. doi: 10.3389/fpubh.2023.1194602 (PMC10359429; doi:10.3389/fpubh.2023.1194602)
Supplement: Supplementary file 1 [file Data_Sheet_1.pdf]

Appendix – Supplementary Material

A Glimpse of Academic Staff Health Behavior on Diet Type and Physical Activity at Austrian Universities: First Findings from the "Sustainably Healthy – From Science 2 Highschool & University" Study

Katharina C. Wirnitzer[1,2,3*], Mohamad Motevalli[1,2], Derrick R. Tanous[1,2], Gerold Wirnitzer[4], Karl-Heinz Wagner[5], Manuel Schätzer[6], Clemens Drenowatz[7], Armando Cocca[2], Gerhard Ruedl[2] and Werner Kirschner[2]

*** Correspondence:**
Katharina C. Wirnitzer
katharina.wirnitzer@ph-tirol.ac.at; Tel.: +43(650)5901794; Web: https://uni.science2.school/en/

Appendix Tables A1 – A4

Table A1: Anthropometric Characteristics by federal state and living area in full-time university staff. Values are means ± SD and prevalence for body weight categories.

	N	Age (years)	Height (cm)	Body Weight (kg)	BMI (kg/m²)	Overweight/ Obesity (%)
**Burgenland**	**18**	**43.0 ± 10.9**	**170.3 ± 6.9**	**73.3 ± 19.6**	**25.2 ± 6.6**	**38.9**
Urban	9	45.0 ±11.0	168.1 ± 6.2	68.7 ± 20.4	24.0 ± 5.6	33.3
Rural	9	41.0 ± 11.0	172.6 ± 7.1	77.8 ± 18.9	26.3 ± 7.6	44.4
**Carinthia**	**8**	**50.1 ± 6.4**	**171.2 ± 9.1**	**71.4 ± 13.8**	**24.0 ± 3.4**	**25.0**
Urban	6	52.5 ± 5.1	168.7 ± 8.1	68.8 ± 15.2	24.0 ± 4.0	33.3
Rural	2	43.0 ± 4.2	181.5 ± 2.1	71.4 ± 4.2	24.0 ± 0.7	0.0
**Lower Austria**	**30**	**48.6 ± 10.4**	**176.3 ± 9.4**	**73.4 ± 15.5**	**23.5 ± 4.2**	**30.0**
Urban	7	51.0 ± 3.5	174.7 ± 7.1	67.5 ± 9.7	22.0 ± 2.1	0.0
Rural	23	47.9 ± 11.7	176.8 ± 10.0	75.2 ± 16.7	24.0 ± 4.6	39.1
**Salzburg**	**36**	**47.8 ± 9.7**	**176.0 ± 9.3**	**76.9 ± 13.3**	**24.8 ± 3.9**	**38.9**
Urban	26	48.0 ± 10.6	175.8 ± 9.9	76.5 ± 14.5	24.7 ± 4.2	34.6
Rural	10	47.2 ± 7.1	176.7 ± 7.7	78.1 ± 9.8	25.1 ± 3.1	50.0
**Styria**	**35**	**49.4 ± 10.4**	**170.9 ± 7.2**	**69.3 ± 9.8**	**23.7 ± 2.8**	**22.9**
Urban	25	51.2 ± 9.7	170.8 ± 7.1	68.8 ± 9.5	23.6 ± 2.6	28.0
Rural	10	44.9 ± 11.5	171.0 ± 7.8	70.4 ± 11.0	24.1 ± 3.2	10.0
**Tyrol**	**189**	**47.3 ± 9.8**	**174.3 ± 9.5**	**73.4 ± 14.1**	**24.1 ± 3.8**	**31.7**
Urban	125	46.7 ± 10.1	173.7 ± 9.9	72.9 ± 13.9	24.1 ± 3.9	31.2
Rural	64	48.5 ± 9.3	175.4 ± 8.5	74.4 ± 14.6	24.0 ± 3.7	32.8
**Upper Austria**	**75**	**49.3 ± 8.8**	**171.9 ± 9.2**	**71.1 ± 13.7**	**24.0 ± 3.6**	**33.3**
Urban	42	48.1 ± 9.8	172.1 ± 8.4	69.9 ± 14.2	23.5 ± 3.8	28.6
Rural	33	50.7 ± 7.5	171.8 ± 10.2	72.7 ± 13.0	24.6 ± 3.4	39.4
**Vienna**	**279**	**44.4 ± 11.3**	**175.8 ± 8.9**	**75.7 ± 13.8**	**24.4 ± 3.4**	**36.6**
Urban	252	44.2 ± 11.3	175.7 ± 8.8	75.7 ± 14.1	24.4 ± 3.4	35.7
Rural	27	46.4 ± 11.3	176.9 ± 9.9	76.2 ± 11.8	24.3 ± 4.0	44.4
**Vorarlberg**	**16**	**50.1 ± 11.4**	**173.3 ± 6.7**	**74.5 ± 16.3**	**24.8 ± 5.1**	**37.5**
Urban	7	51.4 ± 10.7	172.7 ± 7.3	73.8 ± 15.3	24.7 ± 4.4	42.9
Rural	9	49.1 ± 12.4	173.7 ± 6.7	75.1 ± 18.0	24.9 ± 5.8	33.3

Table A2: Anthropometric Characteristics by federal state and living area in part time staff. Values are means ± SD and prevalence for body weight categories.

	N	Age (years)	Height (cm)	Body Weight (kg)	BMI (kg/m²)	Overweight/ Obesity (%)
**Burgenland**	22	45.3 ± 11.6	174.8 ± 10.0	72.7 ± 17.1	23.5 ± 3.4	40.9
Urban	10	46.3 ± 11.5	172.7 ± 9.3	67.1 ± 12.9	22.3 ± 2.6	30.0
Rural	12	44.5 ± 12.1	176.6 ± 10.6	77.4 ± 19.3	24.5 ± 3.7	50.0
**Carinthia**	4	51.7 ± 4.6	161.3 ± 1.2	75.2 ± 32.3	29.0 ± 12.9	50.0
Urban	4	51.7 ± 4.6	161.3 ± 1.2	75.2 ± 32.3	29.0 ± 12.9	50.0
Rural	N / A	N / A	N / A	N / A	N / A	N / A
**Lower Austria**	8	49.1 ± 9.8	168.8 ± 8.6	70.9 ± 12.5	25.0 ± 4.6	37.5
Urban	3	53.0 ± 11.5	166.7 ± 1.2	70.0 ± 17.1	25.1 ± 5.8	33.3
Rural	5	46.8 ± 9.1	170.0 ± 11.1	71.4 ± 11.3	24.8 ± 4.6	40.0
**Salzburg**	31	48.6 ± 11.2	172.5 ± 8.9	71.1 ± 14.4	23.9 ± 4.3	29.0
Urban	24	49.8 ± 11.9	171.8 ± 8.5	70.5 ± 14.6	23.7 ± 3.8	33.3
Rural	7	44.4 ± 7.2	174.7 ± 10.4	73.4 ± 14.8	24.3 ± 6.3	14.3
**Styria**	26	47.3 ± 10.1	169.9 ± 8.4	67.0 ± 12.4	23.0 ± 3.9	30.8
Urban	19	48.0 ± 10.6	169.4 ± 8.0	66.8 ± 12.6	23.1 ± 4.3	31.6
Rural	7	45.4 ± 9.4	171.4 ± 9.9	67.7 ± 12.3	22.8 ± 2.8	28.6
**Tyrol**	92	42.3 ± 13.2	171.4 ± 8.3	68.6 ± 11.2	23.3 ± 3.8	26.1
Urban	49	39.5 ± 13.7	170.7 ± 7.8	67.0 ± 11.9	22.9 ± 3.5	20.4
Rural	43	45.5 ± 11.9	172.2 ± 8.8	70.3 ± 11.0	23.8 ± 4.1	32.6
**Upper Austria**	45	48.2 ± 10.1	171.9 ± 7.9	70.0 ± 12.8	23.6 ± 3.2	26.7
Urban	21	46.9 ± 9.8	170.9 ± 8.1	70.2 ± 14.6	23.9 ± 3.8	33.3
Rural	24	49.3 ± 10.4	1172.8 ± 7.7	69.9 ± 11.3	23.3 ± 2.7	20.8
**Vienna**	123	47.2 ± 15.0	174.7 ± 8.3	72.0 ± 14.2	23.4 ± 3.5	26.8
Urban	112	47.3 ± 15.2	174.8 ± 8.2	72.0 ± 14.1	23.4 ± 3.5	25.9
Rural	11	46.0 ± 13.7	173.8 ± 9.6	72.1 ± 15.7	23.6 ± 3.5	36.4
**Vorarlberg**	4	53.5 ± 11.8	173.5 ± 12.0	79.3 ± 15.8	26.1 ± 2.6	50.0
Urban	N / A	N / A	N / A	N / A	N / A	N / A
Rural	4	53.5 ± 11.8	173.5 ± 12.0	79.3 ± 15.8	26.1 ± 2.6	50.0

Table A3: Health behavior by federal state and living area in full time staff. Values are prevalences (%) as well as mean ± SD for days with PA – physical activity, sports & exercise.

	N	Leisure time PA (%)	Club sports (%)	PA/Sport days per week* (mean±SD)	Daily Fruits (%)	Daily Veggies (%)	Fluid intake >2L/day (%)	Water as most common fluid (%)	Vegetarian /Vegan (%)	Alcohol (%)	Smoking (%)
**Burgenland**	**18**	**88.9**	**16.7**	**3.3 ± 1.1**	**50.0**	**77.8**	**55.6**	**72.2**	**16.7**	**72.2**	**22.2**
Urban	9	88.9	11.1	3.5 ±1.1	55.6	88.9	55.6	67.7	22.2	66.7	22.2
Rural	9	88.9	22.2	3.1 ± 1.1	44.4	66.7	55.6	77.8	11.1	77.8	22.2
**Carinthia**	**8**	**100**	**25.0**	**3.9 ± 1.9**	**87.5**	**62.5**	**62.5**	**62.5**	**12.5**	**50.0**	**0.0**
Urban	6	100	33.3	3.2 ± 1.5	83.3	83.3	50.0	66.7	16.7	33.3	0.0
Rural	2	100	0.0	6.0 ± 1.4	100	0.0	100	50.0	0.0	100	0.0
**Lower Austria**	**30**	**83.3**	**26.7**	**3.9 ± 2.1**	**66.7**	**70.0**	**40.0**	**76.7**	**6.7**	**60.0**	**16.7**
Urban	7	85.7	28.6	4.3 ± 1.6	71.4	42.9	42.9	71.4	0.0	57.1	14.3
Rural	23	82.6	26.1	3.7 ± 2.2	65.2	78.3	39.1	78.3	8.7	60.9	17.4
**Salzburg**	**36**	**80.6**	**8.3**	**3.4 ± 1.6**	**61.1**	**77.8**	**41.7**	**75.0**	**13.9**	**72.2**	**5.6**
Urban	26	80.8	**7.7**	3.2 ± 1.6	57.7	76.9	38.5	76.9	11.5	76.9	7.7
Rural	10	80.0	10.0	3.9 ± 1.6	70.0	80.0	50.0	70.0	20.0	60.0	0.0
**Styria**	**35**	**80.0**	**25.7**	**3.5 ± 1.5**	**68.6**	**82.9**	**40.0**	**82.9**	**17.1**	**71.4**	**5.7**
Urban	25	76.0	24.0	3.6 ± 1.3	68.0	80.0	44.0	80.0	16.0	68.0	4.0
Rural	10	90.0	30.0	3.2 ± 1.8	70.0	90.0	30.0	90.0	20.0	80.0	10.0
**Tyrol**	**189**	**92.6**	**19.6**	**3.1 ± 1.6**	**63.5**	**79.9**	**36.0**	**72.5**	**12.7**	**73.5**	**13.2**
Urban	125	92.0	20.0	3.0 ± 1.6	65.6	75.2	36.8	72.8	14.4	73.6	11.2
Rural	64	93.8	18.8	3.3 ± 1.6	59.4	89.1	34.4	71.9	9.4	73.4	**17.2**
**Upper Austria**	**75**	**90.7**	**20.0**	**3.1 ± 1.5**	**65.3**	**82.7**	**44.0**	**70.7**	**13.4**	**66.7**	**9.3**
Urban	42	92.9	14.3	3.1 ± 1.6	66.7	85.7	38.1	76.2	16.7	69.0	11.9
Rural	33	87.9	27.3	3.1 ± 1.0	63.6	78.8	51.5	63.6	9.1	63.6	6.1
**Vienna**	**279**	**88.9**	**20.1**	**3.4 ± 2.0**	**60.6**	**81.4**	**43.7**	**75.6**	**15.8**	**74.6**	**14.0**
Urban	252	88.9	19.0	3.4 ± 1.9	61.5	81.0	44.4	77.8	16.7	74.6	15.5
Rural	27	88.9	29.6	2.9 ± 2.9	51.9	85.2	37.0	55.6	7.4	74.1	0.0
**Vorarlberg**	**16**	**87.5**	**12.5**	**3.4 ± 1.3**	**50.0**	**75.0**	**31.3**	**87.5**	**18.8**	**62.5**	**6.3**
Urban	7	85.7	14.3	3.2 ± 0.4	42.9	71.4	14.3	71.4	14.3	71.4	0.0
Rural	9	88.9	11.1	3.6 ± 1.7	55.6	77.8	44.4	100	22.2	55.6	11.1

* only participants who reported regular PA/sports participation were included.

Table A4: Health behavior by federal state and living area part time staff. Values are prevalences (%) as well as mean ± SD for days with PA – physical activity, sports & exercise.

	**N**	**Leisure time PA (%)**	**Club sports (%)**	**PA/Sport days per week* (mean±SD)**	**Daily Fruits (%)**	**Daily Veggies (%)**	**Fluid intake >2L/day (%)**	**Water as most common fluid (%)**	**Vegetarian /Vegan (%)**	**Alcohol (%)**	**Smoking (%)**
**Burgenland**	**22**	**90.9**	**22.7**	**2.8 ± 0.9**	**63.6**	**68.2**	**40.9**	**72.7**	**9.1**	**59.1**	**9.1**
Urban	10	80.0	20.0	3.0 ± 0.8	80.0	80.0	20.0	70.0	10.0	50.0	10.0
Rural	12	100	25.0	2.6 ± 0.9	50.0	58.3	58.3	75.0	8.3	66.7	8.3
**Carinthia**	**4**	**75.0**	**0.0**	**3.3 ± 1.5**	**75.0**	**75.0**	**0.0**	**50.0**	**0.0**	**50.0**	**0.0**
Urban	4	75.0	0.0	3.3 ± 1.5	75.0	75.0	0.0	50.0	0.0	50.0	0.0
Rural	N / A	N / A	N / A	N / A	N / A	N / A	N / A	N / A	N / A	N / A	N / A
**Lower Austria**	**8**	**62.5**	**25.0**	**3.2 ± 2.7**	**50.0**	**87.5**	**37.5**	**62.5**	**12.5**	**75.0**	**12.5**
Urban	3	33.3	0.0	5.0 ± N/A	66.7	66.7	66.7	66.7	33.3	66.7	0.0
Rural	5	80.0	40.0	2.8 ± 2.9	40.0	100	20.0	60.0	0.0	80.0	20.0
**Salzburg**	**31**	**87.1**	**6.5**	**3.6 ± 1.7**	**58.1**	**80.6**	**32.3**	**71.0**	**22.6**	**74.2**	**16.1**
Urban	24	83.3	4.2	3.8 ± 1.8	62.5	79.2	25.0	70.8	25.0	70.8	20.8
Rural	7	100	14.3	2.9 ± 1.2	42.9	85.7	57.1	71.4	14.3	85.7	0.0
**Styria**	**26**	**88.5**	**23.1**	**2.8 ± 1.3**	**57.7**	**80.8**	**34.6**	**73.1**	**19.2**	**65.4**	**7.7**
Urban	19	94.7	26.3	2.8 ± 1.4	52.6	78.9	42.1	84.2	10.5	68.4	5.3
Rural	7	71.4	14.3	2.6 ± 0.5	71.4	85.7	14.3	42.9	42.9	57.1	14.3
**Tyrol**	**92**	**94.6**	**20.7**	**3.6 ± 1.5**	**69.6**	**85.9**	**33.7**	**79.3**	**22.8**	**79.3**	**12.0**
Urban	49	93.9	20.4	3.7 ± 1.4	69.4	87.8	32.7	83.7	24.5	81.6	14.3
Rural	43	95.3	**20.9**	3.5 ± 1.5	69.8	83.7	34.9	74.4	20.9	76.7	9.3
**Upper Austria**	**45**	**84.4**	**17.8**	**3.5 ± 1.5**	**66.7**	**77.8**	**26.7**	**80.0**	**11.1**	**84.4**	**20.0**
Urban	21	85.7	14.3	3.6 ± 1.4	71.4	81.0	28.6	76.2	9.5	81.0	19.0
Rural	24	83.3	20.8	3.4 ± 1.6	62.5	75.0	25.0	83.3	12.5	87.5	20.8
**Vienna**	**123**	**83.7**	**12.2**	**3.3 ± 1.5**	**58.5**	**74.8**	**33.3**	**65.0**	**15.5**	**78.9**	**17.1**
Urban	112	83.9	12.5	3.3 ± 1.5	58.9	73.2	33.9	65.2	16.1	80.4	18.8
Rural	11	81.8	9.1	2.7 ± 1.6	54.5	90.9	27.3	63.6	9.1	63.6	0.0
**Vorarlberg**	**4**	**100**	**50.0**	**3.8 ± 2.2**	**25.0**	**25.0**	**0.0**	**50.0**	**0.0**	**75.0**	**0.0**
Urban	N / A	N / A	N / A	N / A	N / A	N / A	N / A	N / A	N / A	N / A	N / A
Rural	4	100	50.0	3.8 ± 2.2	25.0	25.0	0.0	50.0	0.0	75.0	0.0

* only participants who reported regular PA/sports participation were included.
